# Personality judgments from everyday images of faces

**DOI:** 10.3389/fpsyg.2015.01616

**Published:** 2015-10-27

**Authors:** Clare A. M. Sutherland, Lauren E. Rowley, Unity T. Amoaku, Ella Daguzan, Kate A. Kidd-Rossiter, Ugne Maceviciute, Andrew W. Young

**Affiliations:** ^1^Department of Psychology, University of YorkYork, UK; ^2^ARC Centre of Excellence in Cognition and its Disorders, School of Psychology, University of Western AustraliaCrawley, WA, Australia

**Keywords:** person perception, first impressions, face perception, personality, ambient images

## Abstract

People readily make personality attributions to images of strangers' faces. Here we investigated the basis of these personality attributions as made to everyday, naturalistic face images. In a first study, we used 1000 highly varying “ambient image” face photographs to test the correspondence between personality judgments of the Big Five and dimensions known to underlie a range of facial first impressions: approachability, dominance, and youthful-attractiveness. Interestingly, the facial Big Five judgments were found to separate to some extent: judgments of openness, extraversion, emotional stability, and agreeableness were mainly linked to facial first impressions of approachability, whereas conscientiousness judgments involved a combination of approachability and dominance. In a second study we used average face images to investigate which main cues are used by perceivers to make impressions of the Big Five, by extracting consistent cues to impressions from the large variation in the original images. When forming impressions of strangers from highly varying, naturalistic face photographs, perceivers mainly seem to rely on broad facial cues to approachability, such as smiling.

## Introduction

By 2013, Facebook had over 1.23bn monthly active users (Sedghi, 2014) and LinkedIn had over 178 million monthly active users (Quantcast, 2014). Moreover, it is estimated that half of British adults currently searching for a relationship have used online dating (YouGov, 2014). Since each of these types of online experience frequently involves seeing photographs of strangers' faces and forming impressions of the people depicted, it would be useful to understand how first impressions are derived from facial photographs. This is especially important given the real-life consequences of such first impressions. For example, impressions of trustworthiness from facial photographs predict online financial lending decisions (Duarte et al., 2012; Yang, 2014), facial impressions of competence predict voting choices (Todorov et al., 2005; Antonakis and Dalgas, 2009), and facial impressions of attractiveness affect hiring and promotions (Gilmore et al., 1986; Lutz, 2010; Hochschild and Borch, 2011).

Recently, researchers have started to model the structure underlying facial first impressions. In particular, Oosterhof and Todorov (2008) used a principal components analysis to reduce trait judgments made to images of faces into two dimensions. The first dimension corresponded most closely to trustworthiness judgments, and seemed to be particularly driven by emotional expression. The second dimension corresponded most closely to dominance judgments, and seemed to be derived from cues of facial maturity, masculinity, and strength (Oosterhof and Todorov, 2008; see also Walker and Vetter, 2009). Since then, Sutherland and colleagues replicated the approachability (trustworthiness) and dominance dimensions using a large sample of naturally varying images of faces (Sutherland et al., 2013). With this more varied set of faces, Sutherland et al. (2013) also found another dimension they called “youthful-attractiveness” which seemed to correspond to perceptions of decreasing beauty along with associated age (see Todorov et al., 2015 for a recent review of the facial first impressions literature).

While these models of facial first impressions are based on more than just personality judgments (for example, attractiveness, gender or age: Oosterhof and Todorov, 2008; Walker and Vetter, 2009; Sutherland et al., 2013), many of the traits used to create the models can be considered to be what Allport and Odbert (1936) called “pure” personality traits; for example responsibility, extraversion or confidence (Oosterhof and Todorov, 2008; Walker and Vetter, 2009; Sutherland et al., 2013). It seems intuitively likely that people will make these kinds of personality judgments from facial photographs, along with other social judgments, and research on spontaneous descriptions given to faces has indeed found this to be the case (Oosterhof and Todorov, 2008; Sutherland et al., 2015).

However, outwith the field of facial first impressions, the leading model of the structure of personality traits is the Big Five model (see Goldberg, 1993; John and Srivastava, 1999 for reviews). This describes human personality in terms of five dimensions; extraversion, agreeableness, openness (sometimes called intellect: Goldberg, 1990), neuroticism (sometimes contrasted to emotional stability), and conscientiousness (McCrae and Costa, 1987; Goldberg, 1990). The Big Five model applies to both self and peer ratings (see Goldberg, 1993; John and Srivastava, 1999 for reviews), and a number of studies now have looked at judgments of strangers on the Big Five personality dimensions from face photographs, or photographs with minimal target information; mostly entirely to examine the accuracy of these judgments (Watson, 1989; Penton-Voak et al., 2006; Little and Perrett, 2007; Beer and Watson, 2008; Naumann et al., 2009; Back et al., 2010; Kramer and Ward, 2010, 2011; Ivcevic and Ambady, 2012; Jones et al., 2012; Leikas et al., 2013). This raises the question of how these Big Five personality characteristics relate to the broad factors of facial first impressions, since these two literatures have not been integrated.

This lack of cross-talk between the personality psychology and facial impressions literatures might have resulted in part because most studies investigating facial impressions of the Big Five focus on the validity of these impressions. For example, studies have investigated the correspondence between perceptions of the Big Five from real or average faces and actual self-rated Big Five personality scores (e.g., Penton-Voak et al., 2006; Little and Perrett, 2007; Kramer and Ward, 2010, 2011; Jones et al., 2012). These studies have found that there might be a “kernel of truth” to the validity of facial judgments of the Big Five, with above-chance agreement found especially for judgments of extraversion, and often also for agreeableness and neuroticism (Penton-Voak et al., 2006; Little and Perrett, 2007; Kramer and Ward, 2010, 2011; Jones et al., 2012). In terms of the cues involved, perceivers seem to rely on cues to masculinity, age and attractiveness to make these judgments (Little and Perrett, 2007), and internal facial features seem especially influential (Kramer and Ward, 2010). Interestingly, judgments do not just depend on an attractiveness halo effect, since accuracy remains above chance when attractiveness is controlled (Penton-Voak et al., 2006; Little and Perrett, 2007; Kramer and Ward, 2010).

These studies on the Big Five, whose focus has been on the validity of Big Five facial judgments, are usually characterized by the use of carefully controlled face stimuli. For example, studies often employ standardized images of young adult faces taken under laboratory conditions (e.g., frontal-facing, expressionless images: e.g., Penton-Voak et al., 2006) or face average images created from similar standardized stimuli (e.g., Little and Perrett, 2007; Kramer and Ward, 2010). A highly controlled approach is useful to investigate the validity of facial perceptions of the Big Five dimensions of personality, as it allows subtle differences to be isolated between the faces of targets who score high or low on these personality dimensions. However, it leaves open the question of how perceivers judge facial personality when viewing more naturalistic, highly varying face images, similar to the kinds of facial images that one might see while browsing online (i.e., “ambient face images”: Jenkins et al., 2011). This is crucial, because, as described in the beginning of this introduction, we are often exposed to facial images online and the impressions these create can have quite far-reaching consequences. Of course, the face images found online are usually not standardized in the ways typical of most laboratory studies. Yet, only a couple of studies have used unstandardized photographs to investigate the validity of personality impressions from faces, by examining how accurate impressions of the Big Five are when judged from Facebook facial images (Back et al., 2010; Ivcevic and Ambady, 2012). These two studies found that the Big Five were accurately judged (except for neuroticism), and extraversion was especially accurately judged.

More importantly, since these previous studies have concentrated on the accuracy of facial impressions of the Big Five personality dimensions, there has not yet been an investigation of how impressions of the Big Five relate to the models of facial first impressions built from a wider range of attributes, as described at the beginning of the introduction (cf. Oosterhof and Todorov, 2008; Walker and Vetter, 2009). What is currently missing from either field is an approach that tests the correspondence between Big Five personality judgments made from faces with the dimensions of general facial first impressions (trustworthiness, dominance, and youthful-attractiveness) identified in the facial first impressions literature. Indeed, Penton-Voak et al. (2006) raised a similar point in their original work on facial impressions of the Big Five, arguing that future studies need to consider how Big Five judgments relate to general dimensions of facial impressions. Here, we set out to examine this for the first time, by establishing the correspondence between judgments of the Big Five with models from the facial first impressions literature. In order to do this, we utilized a set of 1000 naturally varying face images, the largest set of face images which has been used to investigate impressions of personality so far. This investigation is now especially timely with the more widespread interest in models of facial first impressions (see Todorov et al., 2015 for a recent annual review).

Here, we test how perceivers make personality judgments of the Big Five when given highly varying, naturalistic face photographs (“ambient images”: see Jenkins et al., 2011), and how these Big Five judgments might relate to the dimensions of judgment identified by the facial first impressions literature. Unlike previous studies of facial judgments of the Big Five, we deliberately concentrate here on perceptions rather than examining the extent to which these judgments are accurate. In Brunswik's (1956) terms, we are specifically interested in *cue utilization* rather than *cue validity*.

We set out to examine these questions using a database of 1000 ambient images (photographs) of unfamiliar faces. In Study 1, we had these face images rated on the Big Five dimensions, and examined how these Big Five personality judgments correlated with the approachability (trustworthiness), dominance, and youthful attractiveness factors previously identified in the same set of face images by Sutherland et al. (2013). It is important to emphasize that we are not seeking to test whether or not the Big Five dimensions exist as an alternative structure for forming first impressions of faces. Instead, here we evaluate whether people can agree on their judgements of the Big Five dimensions from a much larger and more varied sample of faces than used in previous work, and if so, how these judgments relate to dimensions arising from the facial first impression literature.

In Study 2, we created average images from faces that were rated high or low on each Big Five dimension in Study 1. Averaging a set of face photographs is a means of emphasizing the cues that were consistently present in the original images (Penton-Voak et al., 2006). Here, averaging allows us to visualize which attributes from the original naturalistic images consistently cue personality judgments. Importantly, this also enabled us to cross-validate these personality impressions with an independent group of participants. Finally, we then quantified the facial attributes that changed along with perceptions of the Big Five in the original face photographs.

## Study 1

### Methods 1

#### Stimuli 1

The stimuli used in Study 1 were a set of 1000 highly varied “ambient image” face photographs used in previous studies (Santos and Young, 2005, 2008, 2011; Sutherland et al., 2013; Vernon et al., 2014). The concept of ambient images emphasizes the importance of the variability between images of faces, such as the kinds of face images we see every day on the internet (Burton et al., 2011; Jenkins et al., 2011). In order to represent this variability and thus allow us to examine natural first impressions, the ambient image database consists of photographs of 500 male and 500 female adult faces taken from the internet. These images were sampled from the internet over a period of 5 years (2000–2005) and were collected by running internet searches for neutral search terms (e.g., “face,” “person”). Internet dating sites and professional websites were also searched to ensure that a wide range of contexts were sampled from. These images are intentionally allowed to vary naturally on many potential cues to impressions, such as pose, head tilt, expression, lighting, and facial paraphernalia such as make-up, hairstyles and glasses, and were tightly cropped around the head and shoulders (Santos and Young, 2005, 2008, 2011; Sutherland et al., 2013; see Figure S2 in Vernon et al., 2014 for an example of these sorts of images). Since cross-cultural or own-race biases were not the focus of this investigation, only faces of Caucasian appearance were used. By using such a large sample of face images, we intended to simulate the everyday experience of walking through a town and seeing the faces of many strangers walk by; or browsing online on social media.

#### Participants and procedure 1

Fifty participants (mean age: 21.7 years, 25 female) were tested in accordance with procedures that were approved by the Ethics Committee of the Psychology Department, University of York. Ten participants each rated 1000 faces on one of the Big Five dimensions (extraversion, agreeableness, openness to experience, neuroticism, or conscientiousness). We chose this task of having participants directly rate the Big Five since we wanted to directly assess how perceivers explicitly make judgments of these aspects of personality; and because this is the task used by previous face perception studies on the Big Five (Penton-Voak et al., 2006; Little and Perrett, 2007). On average, participants took around an hour to complete this task and spent 2.70 s on each face; this is broadly comparable to previous studies on facial first impressions (e.g., Rule et al., 2009). Sample size was determined beforehand and was based on previous research with these stimuli (Sutherland et al., 2013). We chose to focus on college-age students because this matches other face perception studies of personality (Penton-Voak et al., 2006; Back et al., 2010; Ivcevic and Ambady, 2012) and facial first impressions research (Oosterhof and Todorov, 2008). To assist the raters, they were given a description of the appropriate dimension adapted from Wikipedia and including labels taken from the Big Five Inventory (John et al., 1991) and the 10 Item Personality Inventory (Gosling et al., 2003; see the Supplementary Materials). This was considered important since the Big Five dimensions would not necessarily be familiar to our participants, and because each of the Big Five encompasses multiple facets (e.g., openness to experience includes both curiosity and originality).

Participants were tested in a quiet room on a laptop or PC running PsychoPy version 1.76 (Peirce, 2008). Faces appeared in random order with a rating scale underneath. Participants were instructed to rate the faces on a scale of 1–7 for the appropriate Big Five dimension, with the scale labeled on screen (low/high) as not at all/very extraverted, agreeable, open to experience, neurotic or conscientious. The participant pressed the number key that corresponded with their rating of each face, and the next face photograph then appeared after a blank interval of approximately 750 ms. Face photographs were all resized to 150 pixels in height (approximately 5 cm) and varied in width to preserve the original aspect ratio. Participants were given as much time as they wanted to look at each face, but were encouraged to go with their “gut instinct” (Todorov et al., 2005). They first saw 10 faces, randomly drawn from the database, as a practice.

### Results 1

The ratings of the Big Five dimensions showed good inter-rater reliability across participants, with all Cronbach's alphas above 0.7 (openness to experience: α = 0.83, extraversion: α = 0.85, agreeableness: α = 0.78, neuroticism: α = 0.76, and conscientiousness: α = 0.77, all *p* < 0.001; note that the participants are examined as if they are items, in keeping with other face perception literature e.g., Oosterhof and Todorov, 2008). These inter-rater reliabilities demonstrate an underlying core of agreement between the evaluation of each trait by different participants, even though participants were judging highly varying, naturalistic photographs. Since the interrater reliabilities were high, this allowed us to average over the individual participants' ratings and the rest of the analyses were therefore carried out at the level of the faces. Before any other analyses, we reversed the neuroticism ratings, to present them as an evaluation of “emotional stability.” This was done for simplicity, because otherwise the neuroticism scale runs in the reverse direction to the other four scales, with high levels of perceived neuroticism receiving a low rating on the scale.

We then examined the inter-correlations between the Big Five ratings (see Table 1). As an exploratory step, we initially carried this out for male and female faces separately, but since the results were virtually identical across face gender we only report analyses collapsed across face gender. Table 1 demonstrates that there are high correlations between the perceived extraversion, agreeableness, openness to experience and emotional stability ratings (all *r* above 0.69). Conscientiousness diverges, on the other hand, with lower inter-correlations with the other four Big Five ratings (all *r* ≤ 0.33).

**Table 1:** **Inter-correlations between the Big Five ratings**.

	**Openness**	**Extraversion**	**Agreeableness**	**Emotional stability**	**Conscientiousness**
Openness	–				
Extraversion	0.85^**^	–			
Agreeableness	0.69^**^	0.74^**^	–		
Emotional stability	0.79^**^	0.79^**^	0.75^**^	–	
Conscientiousness	0.09^*^	0.19^**^	0.33^**^	0.20^**^	–

** p < 0.001,

* *p < 0.05, all n = 1000*.

In order to examine how judgments of the Big Five relate to previous models of facial first impressions, we then correlated the Big Five ratings with the factor scores for the approachability, dominance, and youthful attractiveness factors identified by Sutherland et al. (2013). These factors were created by rotating 13 ratings of impressions and entering these into a factor analysis; here we use the factor scores derived from this model using the regression method. The correlations between the factor scores and the current ratings are at the level of the faces (see Table 2, top three rows). The separation between conscientiousness ratings and the other four Big Five ratings can again be seen: conscientiousness correlates significantly more with the dominance factor than the approachability or youthful-attractiveness factors [Steiger's test, both *Z*_(997)_ > 1.99, both *p* < 0.05] while the other Big Five ratings correlate significantly more highly with the approachability factor than the other two factors [see Table 2, top three rows; Steiger's test: all *Z*_(997)_ > 22.00, all *p* < 0.001]. None of the Big Five judgments correlate especially highly with the second youthful-attractiveness factor.

**Table 2:** **Correlations (top three rows) and partial correlations controlling for valence (bottom three rows) between the Big Five ratings with Approachability, Youthful-Attractiveness, and Dominance factor scores (from Sutherland et al., 2013)**.

		**Openness**	**Extraversion**	**Agreeableness**	**Emotional stability**	**Conscientiousness**
Valence uncontrolled	Factor 1: approachability	0.82^**^	0.83^**^	0.86^**^	0.85^**^	0.33^**^
	Factor 2: youth-attract	0.28^**^	0.20^**^	0.25^**^	0.24^**^	0.22^**^
	Factor 3: dominance	0.11^**^	0.18^**^	0.04^**^	0.16^**^	0.41^**^
Valence controlled	Factor 1: approachability	0.59^**^	0.56^**^	0.52^**^	0.58^**^	−0.14^**^
	Factor 2: youth-attract	−0.02	−0.18^**^	−0.16^**^	−0.13^**^	0.04
	Factor 3: dominance	0.01	0.10^*^	−0.15^**^	0.06	0.38^**^

** p < 0.001,

* *p < 0.05, all n = 1000*.

We also repeated this analysis while controlling for the overall positivity or negativity of the first impression of the faces (See Table 2; last three rows) using valence ratings on a 1–7 scale (with 1 corresponding to a very negative impression, 7 to a very positive impression) taken from Sutherland et al. (2015). This additional analysis was conducted in order to ascertain whether our results could be attributed to a simple halo or social desirability effect where people rated faces generating a more positive impression higher on the positive pole of each of the Big Five traits. While the correlations between the facial first impression factors and the Big Five ratings dropped, the pattern and significance of the results for the approachability and dominance dimensions remained largely the same [see Table 2, last three rows; the outcome of Steiger's tests to compare the strength of the trait-factor correlations across the factors remained identical: all *Z*_(997)_ > 9.35, all *p* < 0.001]. This indicates that, while a simple halo or social desirability effect can explain some of the relationship between approachability and the Big Five ratings, such a halo effect cannot entirely account for our main findings. However, a halo effect could account for the small positive relationships between the youthful-attractiveness dimension and the Big Five ratings.

Finally, we carried out regression analyses by first entering the valence of the impressions as a predictor, and then entering the three facial first impressions factors simultaneously as predictors, in order to display the unique relationship between these dimensions and the Big Five trait ratings. For openness, extraversion, agreeableness, and emotionality stability, the largest unique predictor was the approachability factor; for conscientiousness, the largest predictor was the dominance factor (see Table 3).

**Table 3:** *****R***^2^ and unstandardized B for the unique contribution of each of the three (Approachability, Youthful-Attractiveness, and Dominance) factors (from Sutherland et al., 2013) in predicting the current Big Five ratings, after controlling for valence**.

		**Openness**		**Extraversion**		**Agreeableness**		**Emotional stability**		**Conscientiousness**	
		**B**	**St. Err**	**B**	**St. Err**	**B**	**St. Err**	**B**	**St. Err**	**B**	**St. Err**
Step 1	Valence	0.80^**^	0.03	0.95^**^	0.03	0.67^**^	0.02	0.80^**^	0.02	0.41^**^	0.03
Step 2	Approachability	0.92^**^	0.04	0.91^**^	0.04	0.45^**^	0.02	0.73^**^	0.03	−0.15^**^	0.05
	Dominance	0.05^*^	0.02	0.11^**^	0.02	−0.05^**^	0.01	0.06^**^	0.02	0.28^**^	0.02
	Youthful-attractiveness	−0.03	0.02	0.10^**^	0.02	0.06^**^	0.01	0.05^*^	0.02	−0.06^*^	0.02
*R*^2^ change		0.19^**^		0.16^**^		0.10^**^		0.15^**^		0.13^**^	
Total *R*^2^		0.67^**^		0.71^**^		0.76^**^		0.73^**^		0.32^**^	

** p < 0.001,

* *p < 0.05, all n = 1000*.

### Discussion 1

In Study 1, we found that participants could consistently form impressions of the Big Five personality traits from a large set of naturalistic face photographs, such as the kind of photographs that can be viewed while browsing online. We further found that ratings of extraversion, agreeableness, openness to experience, and emotional stability corresponded highly with the first factor (trustworthiness or approachability) found in the facial first impressions literature (e.g., Oosterhof and Todorov, 2008; Walker and Vetter, 2009; Sutherland et al., 2013). However, we found that ratings of conscientiousness corresponded most closely with the dominance factor found in the facial first impressions literature (Oosterhof and Todorov, 2008; Walker and Vetter, 2009; Sutherland et al., 2013). This relationship might be explained by conscientiousness primarily relating to traits that primarily affect task-based skills (i.e., capability), which is theorized to underlie the dominance dimension (Oosterhof and Todorov, 2008; for example, perceived intelligence also loads on this factor: Sutherland et al., 2013). Indeed, conscientiousness reliably predicts performance in the workplace (Barrick and Mount, 1991). The other Big Five traits, meanwhile, may link to how approachable a target seems, since someone who is emotionally stable, agreeable, extraverted and open to new experiences is likely to be easier to approach. This may explain why these ratings correlated highly with the first facial impression factor, which is primarily a judgment of approachability (Oosterhof and Todorov, 2008; Sutherland et al., 2013; Walker and Vetter, 2009). Oosterhof and Todorov (2008) also found that emotional stability correlated with their first trustworthiness factor, as did Walker and Vetter (2009) for extraversion; although neither of these studies systematically included the full set of Big Five dimensions. Finally, in a study investigating whether targets could deliberately convey the Big Five personality dimensions, Leikas et al. (2013) also found that judgments of likeability correlated with personality judgments of extraversion, openness, and emotional stability from images of targets' faces and bodies, and that conscientiousness remained distinct, agreeing with our current results (assuming that likability is similar to approachability). However, they also found that agreeableness did not correlate with likeability, a surprising finding and in contrast to the current results. This is likely due to the posed nature of the photographs in this previous study, since targets could not seemingly pose agreeableness (Leikas et al., 2013).

Interestingly, none of the current Big Five ratings corresponded particularly closely with the youthful-attractiveness factor found by Sutherland et al. (2013). This fits with previous work using tightly controlled facial images, which also found that ratings of the Big Five could not be simply explained by judgments of attractiveness (Penton-Voak et al., 2006). Moreover, this small overlap with the youthful-attractiveness factor seemed to reflect a simple halo effect as these relationships disappeared or even reversed when the overall valence was controlled for (how positive or negative the impression was). In contrast, our main result of relationships between the Big Five and the other two factors remained when we partialled out the overall valence of the faces. This, along with the divergence of the conscientiousness ratings, means that our current findings are not simply due to attractiveness or other social desirability halo effects (e.g., as in Thorndike, 1920; Dion et al., 1972). That is, we believe that the perceivers genuinely tried to make a judgment of personality. Nevertheless, there were strong inter-correlations between the Big Five ratings of emotional stability, openness, extraversion, and agreeableness, suggesting that participants used many of the same or highly correlated cues to judge these attributes. Moreover, these cues likely overlapped with cues to approachability, perhaps indicating that they were using broad and simple cues to judge these personality attributes, such as smiling. This fits well with the theoretical position taken by facial first impression models (Oosterhof and Todorov, 2008; Sutherland et al., 2013), which suggest that perceivers make facial first impressions along a small number of fundamental dimensions, based on broad cues (e.g., facial resemblance to smiling when neutral, or genuine smiling). In Study 2, we created average faces to examine these cues in more detail, as well as to cross-validate the Big Five ratings.

## Study 2

**Figure 1:**
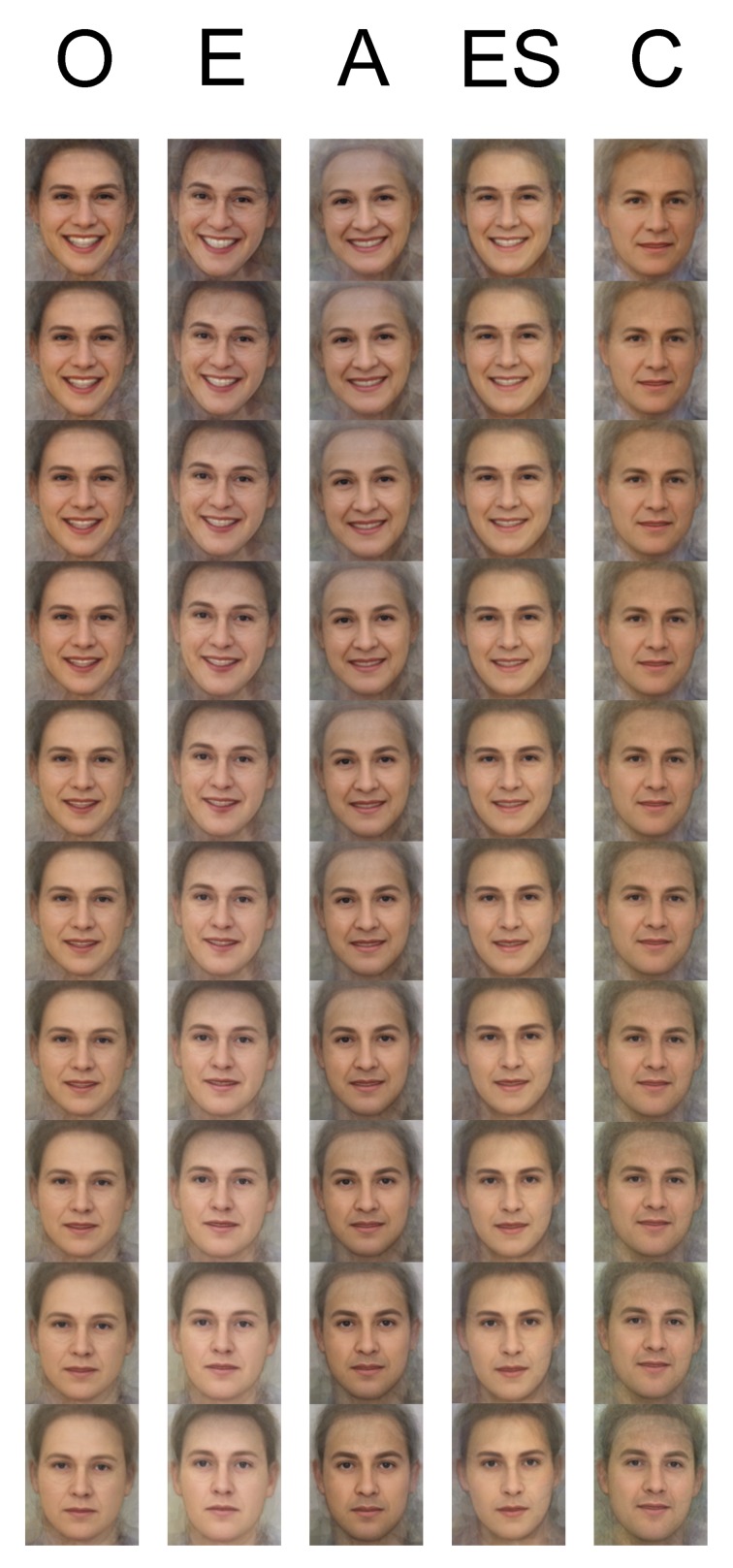
**Linear continua created by morphing in steps of 10% between the high and low Big Five face averages formed from the 20 most and least highly rated faces on the Big Five ratings**. From left to right the columns show openness to experience (O), extraversion (E), agreeableness (A), emotional stability (ES), and conscientiousness (C). The images at the endpoints of each column represent the original average images (high at the top, low at the bottom) for each trait.

In Study 2, we created face-like prototype images by separately averaging the highest and the lowest rated faces for each of the Big Five dimensions. Since averaging leads to cues that are consistent across the individual faces remaining in the average image, this allows us to examine the main cues that participants used to judge the original faces on the Big Five dimensions in Study 1. We then created morphed continua of face images that varied between the low to high prototype for each of the Big Five judgments (see Figure 1). We collected new ratings of the Big Five for each of these continua in order to cross-validate the averaging procedure by demonstrating that the stimuli were perceived as varying on the manipulated Big Five dimension as predicted. This follows procedures used by previous studies on the Big Five (e.g., Penton-Voak et al., 2006; Little and Perrett, 2007), albeit now by averaging together faces high and low on the Big Five in terms of others' perceptions, rather than based on the targets' actual personality scores.

### Methods 2

We created prototype face-like stimuli (see the endpoint images in each row in Figure 1) by averaging together the 20 highest and 20 lowest rated faces for each of the Big Five ratings separately using Psychomorph version 6 (Tiddeman et al., 2001; for a practical guide to averaging procedures in Psychomorph see Sutherland, 2015).

We then created five continua of 11 face images (see Figure 1) that varied between the high and low averaged images for each of the Big Five judgments separately by linearly morphing between the high and low averages using Psychomorph version 6 (Tiddeman et al., 2001; for a practical guide to Psychomorph morphing procedures, see Sutherland, 2015). These images were used to cross-validate the prototypes by testing whether the morphed images were perceived as varying on each Big Five dimension as predicted, using judgments made by a new set of raters.

Ten new participants (mean age = 21.7 years; five female) rated the continua in Study 2. As in Study 1, participants were tested in a quiet room on a laptop or PC running PsychoPy (version 1.76; Peirce, 2008). Participants took around 10 min to complete the task; the average time spent on each face was 3.37 s. Participant sample size was determined beforehand and was based on previous research with similar stimuli (Sutherland et al., 2013). Participants rated images from one continuum at a time, in separate blocks (block order was randomised across participants). Since we were not interested in re-examining the intercorrelations between the Big Five traits, and to prevent carryover effects (Rhodes, 2006), each image continuum was rated only on the manipulated Big Five trait. Within a block, face images appeared in random order with a rating scale underneath; at the beginning of each block the participant first saw all of the faces as practice. Face average images were 400 pixels in height and varied in width to preserve aspect ratio. As in Study 1, participants were given a description of the appropriate Big Five dimension to aid their rating and all other aspects of stimuli presentation were as Study 1.

### Results 2

Figure 1 displays face averages constructed from the highest and lowest scoring faces on the Big Five ratings, allowing an examination of the cues used by participants to make these judgments. The average faces that are high on openness to experience, extraversion, agreeableness, and emotional stability can be seen to be all smiling, whereas their low counterparts look more masculine and more neutral in expression. The high and low face averages for agreeableness in particular look very similar to the high and low approachability face averages created by Sutherland et al. (2013). This agrees with Little and Perrett (2007) who found that average faces created from targets who were low in agreeableness, extraversion and high in neuroticism, were subsequently rated as higher in masculinity than the counterpart average faces. Naumann et al. (2009) also found that observers used smiling as a cue to judge all of the positive poles of the Big Five dimensions from full body photographs. However, the face averages high and low in conscientiousness found here seem to differ in cues other than expression, so that the high conscientious average looks more tanned, clear-skinned and healthy than the low conscientious average (see Figure 1). These conscientiousness averages correspond more to the high and low intelligence face averages depicted in Sutherland et al. (2013).

**Figure 2:**
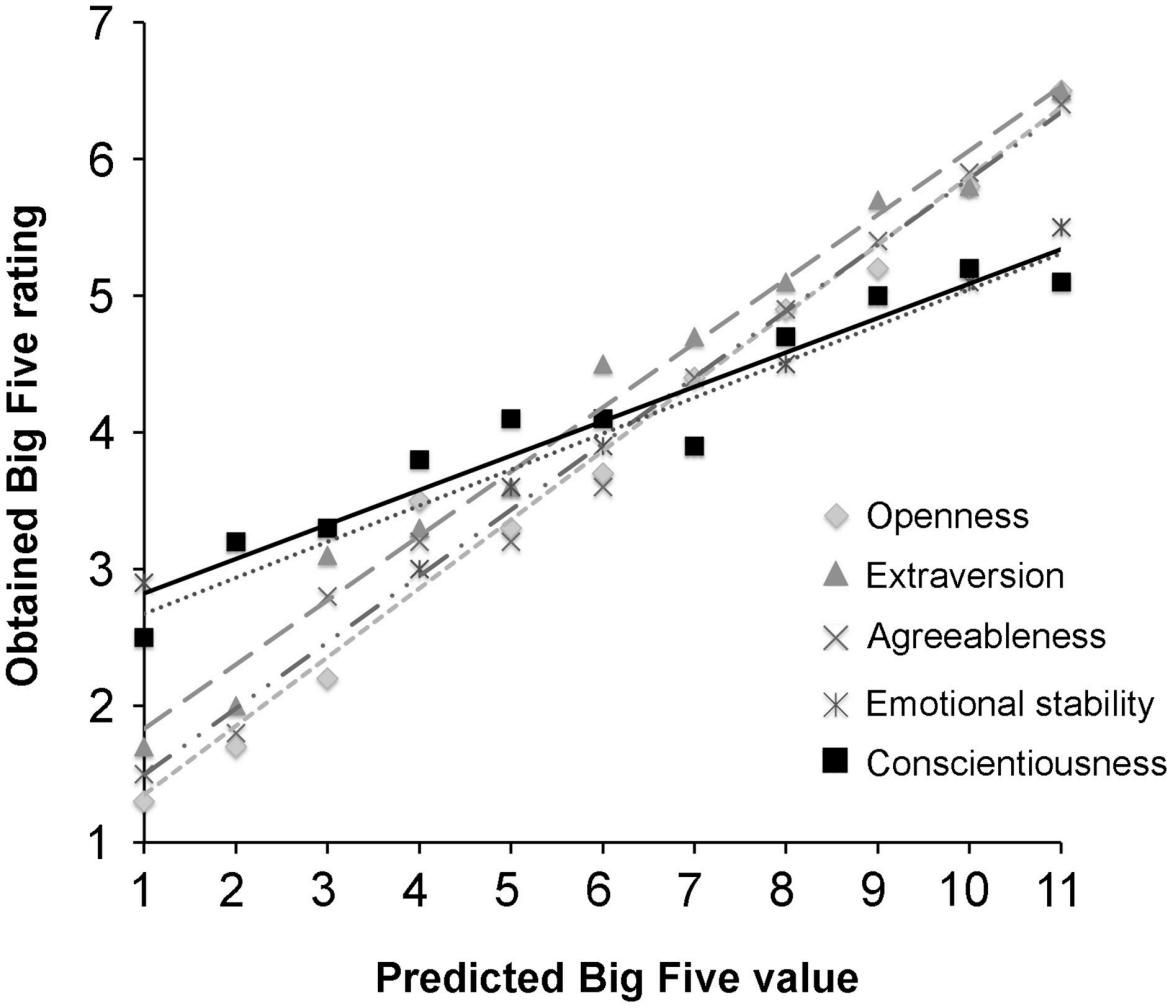
**The manipulated values of the Big Five facial continua plotted against the obtained Big Five ratings**.

In order to cross-validate these stimuli, we morphed between these high and low average faces in steps of 10% (see Figure 1) and had each continuum rated by new participants on the manipulated Big Five dimension. Again, the reliabilities of these new Big Five ratings were all acceptable (all α > 0.7) showing consistency across participants, so we averaged these ratings across participants and then correlated these average ratings with the positions of the stimuli along the generated continuum (i.e., morphing levels 1–11; for a highly similar procedure, see Sutherland et al., 2013). The scatter plots presented in Figure 2 show clear linear relationships, and the aggregated correlation coefficients (see Table 4, first column) are all high (all *r* > 0.95), indicating that on average, participants did view the faces as varying on their respective Big Five personality dimensions as predicted.

**Table 4:** **Correlations between the average obtained Big Five ratings with the predicted Big Five values (i.e., position along each continuum shown in Figure 2), along with the average of the individual correlations between the Big Five ratings with the predicted Big Five values, for the five face continua**.

	**Predicted-obtained**		
	**Aggregate *r***	**Averaged individual *r***	**Standard error mean averaged individual *z'***
Openness	0.99^**^	0.91^**^	0.13
Extraversion	0.99^**^	0.90^**^	0.11
Agreeableness	0.99^**^	0.90^**^	0.08
Emotional stability	0.96^**^	0.60^*^	0.26
Conscientiousness	0.96^**^	0.52^*^	0.17

** p < 0.001,

* *p < 0.01*.

To test that the stimuli faces were perceived as predicted by individual participants, we also correlated each individual participant's rating with the manipulated position of the stimuli, and then averaged across these individual correlations. These averaged (non-aggregated) correlations were lower but still significantly different from zero, indicating that these conclusions were also true at the individual participant level (see Table 4, second column, in which the probabilities are based on comparing the correlation coefficients in one-sample *t*-tests against zero after Fisher's *r*-to-*z* transformation, standard errors for the mean *z*′ corresponding to these tests are shown in the third column).

Finally, to quantify the cues that may have contributed to perceptions of the Big Five, we measured selected cues on the original 1000 faces and entered these attributes into regressions to predict the Big Five ratings (from Study 1). Attributes were measured by marking out points on the face and measuring distances between them, or by measuring the color or texture of the faces (see Vernon et al., 2014 for a detailed description of their computation). Since the extraverted, agreeable, openness, and emotionally stable average faces in Study 2 seemed mainly to have increased in open-mouthed smiling, we selected measurements which might reflect this increase in smiling; including the width of the mouth, the gap between the lips, the curvature of the mouth, and the nose width (i.e., flare of the nostrils). We also measured the height of the mouth, and the distance between the mouth and nose, and size of the eye (iris), since these attributes might be expected to decrease with smiling, as the mouth widens and the eyes crease with smiling. Since the high conscientiousness average face seemed to have darker, more tanned (yellow) and smoother skin than the low conscientiousness average face, we also measured the skin hue, saturation and lightness; as well as how variable (entropic) these were across the face, and for male faces, whether there was stubble present on the face or not. Finally, we included a measure of the steepness of the cheek and eye sockets, since these were significant predictors of the dominance factor (Vernon et al., 2014) and might thus change for conscientiousness given our Study 1 results.

While it is tempting to draw conclusions about the individual predictors, we note that these are naturally multicollinear and thus the predictors are only presented here to give an idea of the overall pattern (see Table 5). In general, we found that structural attributes which increase in open-mouthed smiling (e.g., mouth curvature) highly significantly predicted the openness, extraversion, agreeableness, and emotional stability ratings (see Table 5). Mouth width and mouth-to-nose distance slightly but significantly predicted the conscientiousness ratings, but the open-mouth attributes (gap and height) did not significantly predict conscientiousness. Instead, texture attributes tended to predict conscientiousness, including increasingly dark, yellow, and smoother skin hues (decreasing entropy) and a lack of stubble. Finally, these attribute models predicted more variance in the openness, extraversion, agreeableness and emotional stability ratings (*R*^2^ = 0.55–0.69) than in the conscientiousness ratings (*R*^2^ = 0.19). This is likely due to smiling being such a salient single cue to four of the Big Five, while conscientiousness is perhaps cued by more subtle cues, which combine to create an impression.

**Table 5:** *****R***^2^ and unstandardized B from regressions predicting the Big Five ratings from Study 1 via attributes measured on the faces**.

	**Openness**		**Extraversion**		**Agreeableness**		**Emotional stability**		**Conscientiousness**	
	**B**	**Std. Error**	**B**	**Std. Error**	**B**	**Std. Error**	**B**	**Std. Error**	**B**	**Std. Error**
(Constant)	3.88^**^	0.15	4.42^**^	0.17	3.96^**^	0.13	4.45^**^	0.16	3.47^**^	0.19
Mouth height	0.09	0.14	−0.33^*^	0.15	−0.34^*^	0.12	−0.38^*^	0.14	−0.22	0.18
Mouth width	1.09^**^	0.10	1.28^**^	0.11	0.92^**^	0.09	1.06^**^	0.11	0.33^*^	0.13
Mouth gap	0.49^*^	0.15	0.88^**^	0.17	0.38^*^	0.13	0.43^*^	0.16	−0.08	0.19
Bottom lip curve	1.17^**^	0.11	1.29^**^	0.12	0.62^**^	0.09	0.95^**^	0.12	0.06	0.14
Mouth-nose distance	−0.18^*^	0.08	−0.22^*^	0.09	−0.19^*^	0.07	−0.28^*^	0.08	−0.35^**^	0.10
Nose width	0.29^*^	0.09	0.16	0.1	−0.01	0.07	0.21^*^	0.09	−0.33^*^	0.11
Iris area	−0.21^**^	0.06	−0.24^**^	0.06	−0.04	0.05	−0.31^**^	0.06	0.25^*^	0.07
Skin hue (yellowness-redness)	0.25	0.16	−0.05	0.18	−0.24	0.14	−0.09	0.17	−0.57^*^	0.21
Skin saturation	−0.24^*^	0.12	−0.26^*^	0.13	−0.28^*^	0.10	0.03	0.12	−0.37^*^	0.15
Skin value (brightness)	−0.16^*^	0.06	−0.21^*^	0.07	−0.16^*^	0.05	−0.10	0.06	−0.42^**^	0.08
Hue entropy	−0.36	0.19	−0.78^**^	0.21	−0.99^**^	0.16	−0.50^*^	0.20	−1.77^**^	0.24
Saturation entropy	0.26	0.28	0.84^*^	0.31	0.93^**^	0.24	0.56	0.30	1.57^**^	0.36
Value (brightness) entropy	0.21	0.21	−0.07	0.23	−0.27	0.18	−0.10	0.22	−0.53^*^	0.27
Stubble	0.05	0.03	0	0.03	−0.06^*^	0.02	0.02	0.03	−0.11^*^	0.04
Cheek gradient	0.08	0.09	−0.04	0.10	−0.05	0.07	−0.05	0.09	−0.04	0.11
Eye socket gradient	−0.11	0.08	−0.08	0.09	−0.23^*^	0.07	−0.21^*^	0.08	−0.11	0.10
Eyes-eyebrows distance	0.26^**^	0.07	0.20^*^	0.07	0.4^**^	0.05	0.26^**^	0.07	−0.20^*^	0.08
*R*^2^	0.67^**^		0.69^**^		0.55^**^		0.58^**^		0.19^**^	

** p < 0.001,

* *p < 0.05*.

## General discussion

In the current studies, we investigated how participants judge the Big Five personality dimensions from a diverse, highly variable set of face images, akin to the kinds of images we are exposed to while browsing online. Our intention was to explore *cue utilization* (Brunswik, 1956); that is, to understand which facial cues participants use to make these judgments, regardless of their validity; and how these judgments might relate to dimensions that have been previously identified as important to facial first impressions (Oosterhof and Todorov, 2008; Sutherland et al., 2013). This is the first research to focus on cue utilization for personality judgments from everyday face images.

In Study 1, we found that participants substantially agree on their judgments of the Big Five even to naturalistic face photographs that vary markedly on many characteristics. We also found that the facial judgments of four of the Big Five dimensions were substantially correlated with each other, but that impressions of conscientiousness diverged from impressions of the other Big Five dimensions. In Study 2, we visualized and measured the facial cues underlying these Big Five judgments, finding that smiling seemed to differentiate faces high and low on openness to experience, extraversion, agreeableness, and emotional stability, whereas faces perceived as high in conscientiousness were perhaps more healthy looking than faces perceived as low in conscientiousness, with smoother and more yellow/tanned skin. We were most successful in generating models of facial attributes that could predict the openness to experience, extraversion, agreeableness, and emotional stability judgments, likely due to the saliency of a single cue (smiling) to these judgments. Future research may wish to examine or test other cues to conscientiousness in naturalistic images.

Another key aim was to explore the relationship between facial ratings of the Big Five with the facial first impressions dimensions found in previous work. Interestingly, we found that perceived conscientiousness was related to the dominance factor of facial first impressions found in previous work (Sutherland et al., 2013) whereas judgments of openness, extraversion, emotionality stability, and agreeableness were related to the approachability (trustworthiness) factor. This pattern can be related to Oosterhof and Todorov (2008), who also found that emotional stability correlated with their first factor (trustworthiness); and Walker and Vetter (2009) also found that extraversion correlated with their first, trustworthiness factor.

Our findings also diverge from some previous results. For example, Wiggins' circumplex model of interpersonal traits (Wiggins, 1979) has two dimensions of love and power which seem to correspond well to the facial dimensions of trustworthiness and dominance, respectively (e.g., Oosterhof and Todorov, 2008). With purely verbal stimuli, Wiggins (1996) found that judgments of the agreeableness and extraversion of targets related to the dimensions of love and power, respectively. Beer and Watson (2008) also found that extraversion remained separate from the other Big Five dimensions, which tended to converge in participants' ratings of strangers. This reflects a theoretical distinction made in the personality psychology literature between “alpha” and “beta” factors (Digman, 1997; also called “stability” and “plasticity” factors, respectively; DeYoung, 2006). The alpha factor seems to combine agreeableness, emotional stability, and conscientiousness, while the beta factor combines extraversion and openness (Digman, 1997; DeYoung, 2006).

In the current study, we did not find a strong separation between judgments of facial extraversion and the other Big Five ratings; nor a high correlation between facial extraversion and facial dominance. These differences might be explained by our stimuli, since the current study used photographs of unfamiliar faces whereas previous studies used either familiar targets or strangers viewed in real life. It seems likely that when only given facial photographs, participants will tend to use more broad-based cues to judge traits, such as using a smile as a cue to judge extraversion (see Figure 1). Indeed, this supports Oosterhof and Todorov (2008), who first showed that the majority of variance in facial first impressions can be captured by approachability judgments that rely heavily on cues to emotional expression (Oosterhof and Todorov, 2008; see also Vernon et al., 2014).

Beer and Watson (2008) found that inter-correlations between Big Five judgments were stronger for ratings of strangers (made in real life but with minimal information), than for friends and spouses, and least strong for personality ratings of one's self, indicating that people are more differentiated in their judgments of others they know well compared to judgments of strangers. They suggested that this convergence in Big Five ratings might occur either due to less information or less motivation when judging strangers. We found evidence to support Beer and Watson's (2008) suggestion that judgments of the Big Five should converge as perceivers judge strangers, since we found substantial intercorrelations between judgments of four of the Big Five dimensions made from viewing unfamiliar faces. Future research could test these ideas further for facial first impressions by systematically varying familiarity or motivation and examining if the intercorrelations between perceivers' judgments systematically change, as Beer and Watson's (2008) findings might predict.

Superficially, our finding of a degree of convergence across four of the Big Five dimensions might seem to differ from previous face perception studies using tightly controlled facial stimuli, which have found that judgments of the Big Five from others' faces are partially accurate and that the Big Five dimensions can be separately discriminated (e.g., Penton-Voak et al., 2006; Little and Perrett, 2007). That is, these studies have shown some degree of facial Big Five *cue validity* (Brunswik, 1956). However, our results are actually complementary to this approach, since we designed our studies explicitly to examine facial Big Five *cue utilization*; that is, we examined what cues perceivers use with naturalistically varying stimuli. Our findings should not be taken to suggest that there are no conditions under which perceivers can accurately distinguish between the Big Five. Clearly, previous studies have shown that in fact perceivers can discriminate between the Big Five dimensions when the stimuli constrain differences to subtle but likely critical changes in features (Penton-Voak et al., 2006; Little and Perrett, 2007; Kramer and Ward, 2010) or, for some Big Five judgments, when stimuli are taken from contexts where targets are likely to be deliberately presenting valid cues (Back et al., 2010; Ivcevic and Ambady, 2012). Instead, what we have shown here is that when exposed to a wide range of facial stimuli that differ on many features, perceivers do not necessarily make such fine-grained discriminations, and instead tend to use broad cues such as emotional expression. In addition, participants may also be relying on stereotypes, since previous studies have shown shared semantic content between facial photographs and the content of group stereotypes (Imhoff et al., 2013; Oldmeadow et al., 2013).

Importantly, we also show that this convergence is not fully explained simply by a valence or attractiveness halo: for example, none of the Big Five ratings correlate very highly with a third, youthful-attractiveness factor, especially once valence has been controlled for. This is similar to studies showing that an attractiveness or healthiness halo cannot completely explain the accuracy of facial personality judgments (Penton-Voak et al., 2006; Kramer and Ward, 2010). These results demonstrate the advantages and disadvantages of using everyday, naturalistic face images. On the one hand, one loses the ability to precisely isolate diagnostic cues, as with fine controlled images taken in laboratory conditions (cf. Penton-Voak et al., 2006; Little and Perrett, 2007). On the other hand, one gains the ability to more realistically examine face perception as it might occur in everyday life, with the cues that are realistically available to perceivers (cf. Back et al., 2010; Ivcevic and Ambady, 2012). We therefore view these approaches as complementary.

### Future directions

In the current study we chose to use a college-age sample so that we could draw a parallel between our results and other face perception studies of personality (Penton-Voak et al., 2006; Little and Perrett, 2007; Back et al., 2010; Ivcevic and Ambady, 2012) and impression formation (Oosterhof and Todorov, 2008; Walker and Vetter, 2009; Sutherland et al., 2013). Our participants were also all Caucasian and from a middle-class demographic. In some sense, this may be the ideal sample to start with since these participants are likely social media users, who regularly encounter photographs of strangers in the scenarios outlined in the Introduction (e.g., on Facebook or LinkedIn). However, this also naturally limits the generalizability of our conclusions. In particular, it will be important for future work on facial first impressions to build models of these perceptions that are derived from more inclusive samples from varied cultural and demographic backgrounds than are currently used in this field. A second interesting direction for future work is to examine how photographs taken from different online contexts might lead to different perceptions of personality traits, different relationships between traits or differential validity. For example, company webpages might lead to systematically different representations of conscientiousness or agreeableness than personal websites might. This is quite likely given that different online contexts promote different self-presentation goals (Todorov and Porter, 2014) and that Leikas et al. (2013) have found that targets can deliberately pose to effectively create impressions of the Big Five (except agreeableness). The current photographs were sampled across a wide range of contexts. Similarly, it might be interesting to examine how the context within the photograph may affect perceptions of the face, or whether perceivers have expectations for which faces should appear in which contexts (Todorov and Porter, 2014). Finally, it could also be interesting in future to investigate whether a model of the Big Five can be built using many ratings of faces in a factor analytic approach, following the personality literature (e.g., Goldberg, 1990) and whether this fits better than current two- or three-dimensional facial first impressions models, in part derived from spontaneous impressions of faces (e.g., Oosterhof and Todorov, 2008; Sutherland et al., 2013). This line of work may also be able to investigate whether higher-order factors exist in facial impressions. The current research, by attempting to initiate cross-talk between researchers working in face perception and personality psychology, opens up these kinds of questions as interesting further directions.

## Conclusions

In summary, we show that perceivers can consistently judge highly varying “ambient image” faces in terms of the Big Five personality traits. Moreover, the facial Big Five judgments seem to separate so that openness, extraversion, emotional stability, and agreeableness judgments correspond highly with facial first impressions of approachability, while conscientiousness judgments correspond more with facial first impressions of dominance. When judging everyday images of strangers' faces, perceivers seem to begin by relying on broad and simple cues to overall impressions, such as the presence of a smile.

### Conflict of interest statement

The authors declare that the research was conducted in the absence of any commercial or financial relationships that could be construed as a potential conflict of interest.
