# Supplementary material for: Personality judgments from everyday images of faces
Source: Front Psychol. 2015 Oct 27;6:1616. doi: 10.3389/fpsyg.2015.01616 (PMC4621398; doi:10.3389/fpsyg.2015.01616)
Supplement: Supplementary file 1 [file DataSheet1.DOCX]

***Supplementary Material***

**Personality judgments from everyday images of faces**

**Clare A.M. Sutherland^12*^, Lauren E. Rowley**^1^**, Unity T. Amoaku**^1^**, Ella Daguzan**^1^**, Kate A. Kidd-Rossiter**^1^**, Ugne Maceviciute**^1^**, & Andrew W. Young**^1^

^1^ Department of Psychology, University of York, York, UK.

^2^ ARC Centre of Excellence in Cognition and its Disorders, School of Psychology, University of Western Australia, Crawley, WA 6009, Australia.

* **Correspondence**: Clare Sutherland, School of Psychology, University of Western Australia, Crawley, WA 6009, Australia. Email: clare.sutherland@uwa.edu.au.

**Supplementary Materials**

Descriptions of the Big Five that were given to participants in Study 1 and 2. Descriptions were taken from Wikipedia (Wikipedia, 2013) to ensure they were layman friendly, and contained labels adapted from the Big Five Inventory (John, Donahue & Kentle, 1991; shown here with a double underline) and the Ten Item Personality Inventory (Gosling, Rentfrow & Swann, Jr, 2003; shown here with a single underline). Note that the text was shown to participants without the underlining.

**Openness**

Those open to experience tend to be creative, have an active imagination, and original or inventive in coming up with new ideas. They are complex, ingenious and deep thinkers who like to reflect and play with ideas. They are curious and willing to try new things and are typically interested in art or learning.

A person who is low in openness to experience tends to be conventional, less creative, and traditional. They prefer familiar routines to new experiences, have few artistic interests and tend to prefer the straightforward.

**Conscientiousness**

Those scoring highly on conscientiousness are usually careful, efficient and reliable workers who will make plans and follow these through. They will want to carry a task out well and will persevere until it is finished. They are generally self-disciplined and dependable but at extremes can be perfectionists.

Those who score low on conscientiousness tend to be laid back, less organised, and can be careless or easily distracted. They are less goal orientated or driven and at extremes, can be lazy.

**Extraversion**

Extraverts enjoy interacting with others and tend to be very energetic, talkative and emotionally expressive. These individuals are outgoing, favour spending time in social situations and can be prone to boredom when by themselves. They appear enthusiastic, action orientated and will assert themselves.

Those scoring low on extraversion tend to be quieter and more thoughtful, particularly in social situations. They are generally reserved, can be shy or inhibited and enjoy time spent alone. They are not necessarily unfriendly, but simply prefer solitary activities.

**Agreeableness**

Individuals who score highly on agreeableness are perceived as sympathetic, warm, kind and considerate. They value getting along with others, and are generally helpful and unselfish. They have a trusting, optimistic view of human nature, and usually prefer cooperation or forgiveness than conflict.

Those scoring low on agreeableness tend to be more sceptical about other people's motives, sometimes resulting in criticism, fault-finding, quarrelsomeness or rudeness. They are more likely to compete than to cooperate and can appear cold or aloof.

**Neuroticism**

Individuals who score high on neuroticism get nervous easily and are more likely to experience feelings such as anxiety or a depressed mood. They are also likely to interpret situations as threatening or hopelessly difficult, worry a lot, and can be moody or tense.

Individuals who score low in neuroticism are more emotionally stable, not easily upset and handle stress well. They tend to be calm (even in tense situations), relaxed and even-tempered (although they are not necessarily high on positive emotion).

**References:**

Gosling, S. D., Rentfrow, P. J., & Swann Jr, W. B. (2003). A very brief measure of the Big-Five personality domains. *Journal of Research in Personality*, *37*(6), 504–528.

John, O. P., Donahue, E. M., & Kentle, R. L. (1991). The big five inventory—versions 4a and 54. *Berkeley: University of California, Berkeley, Institute of Personality and Social Research*.

Wikipedia. (2013). Big Five personality traits. Retrieved August 16, 2015, from https://en.wikipedia.org/wiki/Big_Five_personality_traits
